# Supplementary material for: An mRNA-binding channel in the ES6S region of the translation 48S-PIC promotes RNA unwinding and scanning
Source: eLife. 2019 Dec 2;8:e48246. doi: 10.7554/eLife.48246 (PMC6887119; doi:10.7554/eLife.48246)
Supplement: Supplementary file 1. — The predicted secondary structure (RNAfold) is depicted in dot-bracket notation. Stem-loops are marked in red together with the predicted stability (ΔG°). The predicted stability for the entire 5′ UTR is shown, including the correction for 5′ UTR length. For the 5′ UTR of luc mRNAs, shared sequences are in bold. [file elife-48246-supp1.docx]

**Supplementary file 1. Sequence and secondary structure of the 5´UTRs used in study.**

Flat mRNA

GACCCACCAACACAGCACC**AUG**AACAACGAGCCACCGACAGGUGAUGAGUGAUGACGGAGGCACACACGACAGACAACCGAGAGAGCAGAACGAGACCACAC(A)25

......((.......((((((...........((((....))))))).))).....))............................................

DLP 27 mRNA

-27 kcal.mol^-1^

GACCCACCAACACAGCACC**AUG**AACAACGAGCCACCGCAAACCAGUGACGGCCGCCGGAGGCGCCCGCGCCCGGCGGCCGAGAGAGCAGAACGAGACCACAC(A)25

...................................(((......))).((((((((((..((....))..))))))))))......................

5´UTR of luc mRNAs:

5´UTR-33 (-7.2 kcal.mol^-1^, -0.21 kcal.mol^-1^.nt^-1^)

GGAGACGAAUUCGGAUCCGUCGACAGAUCUACC**AUG**

(((..((....))..)))(((....)))........

5´UTR-63(-1 kcal.mol^-1^, -0.01 kcal.mol^-1^.nt^-1^)

**UCAGAUCCGCUAGC**ACCACCAGCCAACCACCUUUUCACCACCACCACCCACCAC**GAUCCAAUG**

........(((.........)))........................................

5´UTR G-less(0 kcal.mol^-1^)

CCACUAUCUCACACCUUUCCUCACUCUUUCCUCACACUUCUUUCUACACUCUUCACAAAAAAUAAUUUCUCACUUCCUAUUCUUCUCCCCCAUCCCUCAUUCCUCAAUCAUUCCUUCCCCAUUCACUUCAAUCAUUCCAA**AUG**

...............................................................................................................................................

5´UTR-85(-37 kcal.mol^-1^, -0.43kcal.mol^-1^.nt^-1^)

**UCAGAUCCGCUAGC**GCUACCGGACUCAGAUCUCGAGCUCAAGCUUCGAAUUCUGCAGUCGACGGUACCGCGGGCCCGG**GAUCCAAAUG**

...((((((((.(((.(((((((((((((..((((((....)).))))..)))).))))..))))).))).)))...)))))......

5´UTR-SL30(-51 kcal.mol^-1^, -0.53 kcal.mol^-1^.nt^-1^) -32 kcal.mol^-1^

**UCAGAUCCGCUAGC**GCUACCGGACUCAGAUCUCGAGCUCAAGCUUCGAAUUCUGCAGUCGACCCGGGCCCGCGGAGUACCGCGGGCCCGG**GAUCCAAUG**

........((....)).....((((((((..((((((....)).))))..)))).))))(((((((((((((((....))))))))))))).)).....

5´UTR-SL50(-67.40 kcal.mol^-1^, -0.62kcal.mol^-1^.nt^-1^) -48 kcal.mol^-1^

**UCAGAUCCGCUAGC**GCUACCGGACUCAGAUCUCGAGCUCAAGCUUCGAAUUCUGCAGUCGACCCGGGCCCGCGGUACGCCGAUAGGCGUACCGCGGGCCCGG**GAUCCAAUG**

........((....)).....((((((((..((((((....)).))))..)))).))))(((((((((((((((((((((....))))))))))))))))))).)).....

5´UTR-SL20(-63.7 kcal.mol^-1^, -0.53 kcal.mol^-1^.nt^-1^) -20 kcal.mol^-1^

**UCAGAUCCGCUAGC**GCUACCGGACUCAGAUCUCGAGCUCAAGCUUCGAAUUCUGCAGUCGACGGUACCGCGGGCCCUCGACCCGGGCCCGCGGUACGCCGAUAGGCGUACGG**GAUCCAAUG**

...(((((((....))(((((((((((((..((((((....)).))))..)))).))))..))))).(((((((((.......)))))))))(((((((....))))))).))))).....

5´UTR-242 (-60.10 kcal.mol^-1^, -0.24 kcal.mol^-1^.nt^-1^)

**UCAGAUCCGCUAGC**GCUACCGGACUCAGAUCUCGAGCUCAAGCUUCGAAUUCUGCAGUCGACGGUACCGCGGGCCCAGGUAGACAAUAUUACACCUGUCCUACUGGCAUUGAGAACUUUUGCCCAGAGCAAAAGAGCAUUCCAAGCCAUCAGAGGGGAAAUAAAGCAUCUCUACGGUGGUCCUAAAUAGUCAGCAUAGUACAUUUCAUCUGACUAAUACUACAACACCACCACCUCUAGA**CCAUG**

.....((((((.(((.(((((((((((((..((((((....)).))))..)))).))))..))))).))).))).(((((............))))).(((..((((.(((.((((((((((.....)))))))....))))))))))....))))))...........((((.((((((......(((((((...............))))))).............))))))..)))).....

5´UTR-427 (-164.70 kcal.mol^-1^, -0.38 kcal.mol^-1^.nt^-1^)

**UCAGAUCCGCUAGC**GCUACCGGACUCAGAUCUCGAGCUCUCUGGCUAACUAGGGAACCCACUGCUUAAGCCUCAAUAAAGCUUGCCUUGAGUGCUUCAAGUAGUGUGUGCCCGUCUGUUGUGUGACUCUGGUAACUAGAGAUCCCUCAGACCCUUUUAGUCAGUGUGGAAAAUCUCUAGCAGUGGCGCCCGAACAGGGACUUGAAAGCGAAAGGGAAACCAGAGGAGCUCAAGCUUCGAAUUCUGCAGUCGACGGUACCGCGGGCCCCUUGGCCUGGCUUCGCUCUACGCCGGCGCGCGCGCGGCGCGAAUUGUAGGUGGGCGUGGCCUCCAAGGGCGUGGCGGCAUUCGUGGUCUCCAUCGCCUGCCAUAAAACACUUGUGUUGGUAGGAAAUCCAUUCUAGAGCGCCCCUAUAGUGGG**GAUCCAAUG**

...((((((((.(((.(((((((((((((..(((((((((((((....)))))))((.(((((((((((((((((...........))))).)))).)))))))).)).........(((.((..(((((((.....(((....)))...(((((((.((((.((((((.....)))).)).)))).(((.....)))..........)))))))..)))))))..)).))))).))))..)))).))))..))))).))).)))(((((((...((((.(((((((((....(((((.....)))))...)))))...)))).)))).)))))))(((((.(((.......))).))).))(((((((....((....))..)))))))....(((((.(((.......))).)))))))))).....

5´UTR-656(-236 kcal.mol^-1^,-0.36kcal.mol^-1^.nt^-1^)

**UCAGAUCCGCUAGC**GCUACCGGACUCAGAUCUCGAGCUCAAGCUUCGAAUUCUGCAGUCGACGGUACCGCGGGCCCAGGUAGACAAUAUUACACCUGUCCUACUGGCAUUGAGAACUUUUGCCCAGAGCAAAAGAGCAUUCCAAGCCAUCAGAGGGGAAAUAAAGCAUCUCUACGGUGGUCCUAAAUAGUCAGCAUAGUACAUUUCAUCUGACUAAUACUACAACACCACCACCUCUAGCGCUACCGGACUCAGAUCUCGAGCUCUCUGGCUAACUAGGGAACCCACUGCUUAAGCCUCAAUAAAGCUUGCCUUGAGUGCUUCAAGUAGUGUGUGCCCGUCUGUUGUGUGACUCUGGUAACUAGAGAUCCCUCAGACCCUUUUAGUCAGUGUGGAAAAUCUCUAGCAGUGGCGCCCGAACAGGGACUUGAAAGCGAAAGGGAAACCAGAGGAGCUCAAGCUUCGAAUUCUGCAGUCGACGGUACCGCGGGCCCCUUGGCCUGGCUUCGCGCUACGCCGGCGCGCGCGCGGCGCGAAUUGUAGGUGGGCGUGGCCUCCAAGGGCGUGGCGGCAUUCGUGGUCUCCAUCGCCUGCCAUAAAACACUUGUGUUGGUAGGAAAUCCAUUCUAGAGCGCCCCUAUAGUGGG**GAUCCAAUG**

...((((((((.(((.(((((((((((((..((((((....)).))))..)))).))))..))))).))).)))((((...((((..........))))...))))..(((.((((((((((.....)))))))....)))))).((((...(((((...........(((((.((((((((((..(((((((...............)))))))......((((((..((((((...(((.(((((((((((((..(((((((((((((....)))))))((.(((((((((((((((((...........))))).)))).)))))))).)).........(((.((..(((((((.....(((....)))...(((((((.((((.((((((.....)))).)).)))).(((.....)))..........)))))))..)))))))..)).))))).))))..)))).))))..))))).)))(((((....)))))...((((((((.(((((...)).))).))))))))....))))))..(((((......((((((((.(((.......))).))).)))))))))).........)))))).)))))...))))).)))))..)))))...))))))))).....

5´UTR-85(-37 kcal.mol^-1^, -0.43kcal.mol^-1^.nt^-1^)

**UCAGAUCCGCUAGC**GCUACCGGACUCAGAUCUCGAGCUCAAGCUUCGAAUUCUGCAGUCGACGGUACCGCGGGCCCGG**GAUCCAAAUG**

...((((((((.(((.(((((((((((((..((((((....)).))))..)))).))))..))))).))).)))...)))))......

5´UTR G4-1 (-71.8 kcal.mol^-1^, -0.8kcal.mol^-1^.nt^-1^) -54 kcal.mol^-1^

**UCAGAUCCGCUAGC**GCUACCGGACUCAGAUCUCGAGCUCAAGCUUCGAAUUCUGCAGGGGUGGGGUGGGGUGGGGCCCGG**GAUCCAAUG**

...(((((((....))...(((...((((..((((((....)).))))..))))..++++.++++.++++.++++.)))))))).....

5´UTR G4-2 (-40.62 kcal.mol^-1^, -0.46kcal.mol^-1^.nt^-1^) -22.8 kcal.mol^-1^

**UCAGAUCCGCUAGC**GCUACCGGACUCAGAUCUCGAGCUCAAGCUUCGAAUUCUGCAGGGAGGGAGGGAGAGGGCCCGG**GAUCCAAUG**

...(((((((....))...(((...((((..((((((....)).))))..))))..+++.+++.+++...+++.)))))))).....

5´UTR G4-3 (-35.4 kcal.mol^-1^, -0.36kcal.mol^-1^.nt^-1^) -11 kcal.mol^-1^

**UCAGAUCCGCUAGC**GCUACCGGACUCAGAUCUCGAGCUCAAGCUUCGAAUUCUGCAGGGGGCCGUGGGGUGGGAGCUGGGGCCCGG**GAUCCAAUG**

...((((((.((....)).)))).)).(((((((.((((.((((((..(((((((.((...))))))))).)))))).)))).))))))).....
