# Supplementary material for: An mRNA-binding channel in the ES6S region of the translation 48S-PIC promotes RNA unwinding and scanning
Source: eLife. 2019 Dec 2;8:e48246. doi: 10.7554/eLife.48246 (PMC6887119; doi:10.7554/eLife.48246)
Supplement: Supplementary file 2. — The sequences of oligonucleotides used for blocking the ES6S region and for PCR amplification are shown. [file elife-48246-supp2.docx]

| **oligo name** | **oligo sequence** | **purpose** |
| --- | --- | --- |
| oligo 3 | CACCTCTAGCGGCGCAATACG | binding to 18S rRNA (973-957)* |
| oligo 4 | CGGTATCCAGGCGGCTCGG | binding to 18S rRNA (830-848)* |
| oligo 5.4 | CGCTTCGGGCCCCGCGGG | binding to 18S rRNA (777-794)* |
| oligo 9 | CGTCACTACCTCCCCG | binding to 18S rRNA (493-508)* |
| oligo C | GCCTGTCCAATGACTAGGGCACTGACGG | negative control |
| fw SFV S-UTP | GGATCCTAATACGACTCACTATAGGGACCCACCAACACAGCACCATGAACAACGA | templates for the synthesis of SFV mRNAs |
| rev S-UTP +0 | GTGTGGTCTCGTTCTGCTCTCTCGGCCGCCGGGCGCGGGCGCCTCCGGCGGCCGTCTCTGGTTTGCGGTGGCTCGTTGTTCATGGTGCTGTGTTG | templates for the synthesis of SFV mRNAs |
| rev S-UTP +12 | GTGTGGTCTCGTTCTGCTCTCTCGGCCGCCGGGCGCGGGCGCCTCCGGCGGCCGTCTCTGGTTTGCGCGTTATCGTTGTTCATGGTGCTGTGTTG | templates for the synthesis of SFV mRNAs |
| rev S-UTP +15 | GTGTGGTCTCGTTCTGCTCTCTCGGCCGCCGGGCGCGGGCGCCTCCGGCGGCCGTCTCTGGTTTGCGTATTCTCGTTGTTCATGGTGCTGTGTTG | templates for the synthesis of SFV mRNAs |
| rev S-UTP +18 | GTGTGGTCTCGTTCTGCTCTCTCGGCCGCCGGGCGCGGGCGCCTCCGGCGGCCGTCTCTGGTTTGATTTGGCTCGTTGTTCATGGTGCTGTGTTG | templates for the synthesis of SFV mRNAs |
| rev S-UTP +21 | GTGTGGTCTCGTTCTGCTCTCTCGGCCGCCGGGCGCGGGCGCCTCCGGCGGCCGTCTCTGGTATTCGGTGGCTCGTTGTTCATGGTGCTGTGTTG | templates for the synthesis of SFV mRNAs |
| rev S-UTP +24 | GTGTGGTCTCGTTCTGCTCTCTCGGCCGCCGGGCGCGGGCGCCTCCGGCGGCCGTCCGTATTGGGCGGTGGCTCGTTGTTCATGGTGCTGTGTTG | templates for the synthesis of SFV mRNAs |
| rev S-UTP +27 | GTGTGGTCTCGTTCTGCTCTCTCGGCCGCCGGGCGCGGGCGCCTCCGGCGGCCGGTATTGGTTTGCGGTGGCTCGTTGTTCATGGTGCTGTGTTG | templates for the synthesis of SFV mRNAs |
| rev S-UTP +30 | GTGTGGTCTCGTTCTGCTCTCTCGGCCGCCGGGCGCGGGCGCCTCCGGCGGCCGATTGCTGGTTTGCGGTGGCTCGTTGTTCATGGTGCTGTGTTG | templates for the synthesis of SFV mRNAs |
| rev S-UTP +33 | GTGTGGTCTCGTTCTGCTCTCTCGGCCGCCGGGCGCGGGCGCCTCCGGCGGCCGATTGTCTCTGGTTTGCGGTGGCTCGTTGTTCATGGTGCTGTGTTG | Templates for the synthesis of SFV mRNAs |
| rev S-UTP +36 | GTGTGGTCTCGTTCTGCTCTCTCGGCCGCCGGGCGCGGGCGCCTCCGGCGGCCGATTGTTGTCTCTGGTTTGCGGTGGCTCGTTGTTCATGGTGCTGTGTTG | Templates for the synthesis of SFV mRNAs |
| fw flat | GGATCCTAATACGACTCACTATAGGGACCCACCAACACAGCACCATGAACAACGAGCCACCGACAGGTGATGAGTGATGACGGAGGCACACACGACAGACAACCGAGAGAGCAGAACGAGACCACAC | Templates for the synthesis of flat mRNA |
| T7-pEGFPN1 | GGATCCTAATACGACTCACTATAGGGGTCAGATCCGCTAGCGCTACCGGAC | Templates for the synthesis of luc mRNAs |
| T7-U-fw | GGATCCTAATACGACTCACTATAGGG | Templates for the synthesis of SFV mRNAs |
| revU_DLP_polyA | (T)25 GTGTGGTCTCGTTCTGCTC | Templates for the synthesis of SFV mRNAs |
| fw-hRPS4X | GCCACCGCTAGCACCATGGCTCGTGGTCCCAAGAAGC | Cloning & expression of RPS4X-EGFP fusion protein |
| rev-hRPS4X | TCGAAGCTTTGGATCCTGCAGCCCACTGCTCTGTTTGGCCGCCAG | Cloning & expression of RPS4X-EGFP fusion protein |
| rev-hRPS4X-stop | CAGTGGGGCCCTCAGTCTAGACTGCTCTGTTTGGCCGCCAGTCTTTTGTC | Cloning & expression of RPS4X |
| fw-St30 | TCGACCCGGGCCCGCGGA | Insertion of SL30 into the  5´UTR luc mRNA |
| rev-St30 | GTACTCCGCGGGCCCGGG | Insertion of SL30 into the  5´UTR luc mRNA |
| fw-St50 | TCGACCCGGGCCCGCGGTACGCCGATAGGC | Insertion of SL50 into the  5´UTR luc mRNA |
| rev-St50 | GTACGCCTATCGGCGTACCGCGGGCCCGGG | Insertion of SL50 into the  5´UTR luc mRNA |
| fw_UTR_G-less | CCACTATCTCACACCTTTCCTCACTCTTTCCTCACACTTCTTTCTACACTCTTCACAAAAAATAATTTCTCACTTCCTATTCTTCTCCCCCATCCCTCATTCCTCAATCATTCCTTCCCC | Construction of G-less 5’UTR luc mRNA |
| rev_UTR_G-less | CTGAGATGAGTTTTTGTTCCATTTGGAATGATTGAAGTGAATGGGGAAGGAATGATTGAGGAATGAGGGATGGGGGAGAAGAATAGGAAGTGAGAAATTATTTTTTGTGAAG | Construction of G-less 5’UTR luc mRNA |
| fw-linker(GGC) | ATCCGGCGGCGGCGGCTCTTCACTGCA | Insertion of (GGC)_4_ into the 5´UTR luc mRNA |
| rev-linker(GGC) | GTGAAGAGCCGCCGCCGCCGGATTGCA | Insertion of (GGC)_4_ into the 5´UTR luc mRNA |
| fw-G4-1 | GGGGTGGGGTGGGGTGGGGCC | Insertion of G4-1 into the 5´UTR luc mRNA |
| rev-G4-1 | CCACCCCACCCCACCCCTGCA | Insertion of G4-1 into the 5´UTR luc mRNA |
| fw-G4-2 | GGGAGGGAGGGAGAGGGCC | Insertion of G4-2 into the 5´UTR luc mRNA |
| rev-G4-2 | CTCTCCCTCCCTCCCTGCA | Insertion of G4-2 into the 5´UTR luc mRNA |
| fw-G4-3 | GGGGGCCGTGGGGTGGGAGCTGGGGCC | Insertion of G4-3 into the 5´UTR luc mRNA |
| rev-G4-3 | CCAGCTCCCACCCCACGGCCCCCTGCA | Insertion of G4-3 into the 5´UTR luc mRNA |
| 3’RTTS_adapter | AGATCGGAAGCGTCGGACTGTAGAACTCTGAACGTGT | RTTS (adapter ligation) |
| 3’_adapter | AGACGTGTGCTCTTCCGATCTCACCTCTAGCGGCGCAATACG | RTTS amplification |
| PR_FWD | ATGATACGGCGACCACCGAGATCTACACGTTCAGAGTTCTACAGTCCGAG | RTTS amplification |
| RP_REV_INDEX5 | CAAGCAGAAGAACGGCATACGAGATACAGTGGTGACTGGAGTTCAGACGTGTGCTCTTCCGATCT | RTTS index crosslinked SFV-DLP 27 mRNA |
| RP_REV_INDEX6 | CAAGCAGAAGAACGGCATACGAGATGCCAATGTGACTGGAGTTCAGACGTGTGCTCTTCCGATCT | RRTS index total SFV-DLP 27 mRNA |
| RP_REV_INDEX7 | CAAGCAGAAGAACGGCATACGAGATCAGATCGTGACTGGAGTTCAGACGTGTGCTCTTCCGATCT | RTTS index crosslinked flat mRNA |
| RP_REV_INDEX12 | CAAGCAGAAGAACGGCATACGAGATCTTGTAGTGACTGGAGTTCAGACGTGTGCTCTTCCGATCT | RTTS index total flat mRNA |
